# Energy Efficient Moving Target Tracking in Wireless Sensor Networks

**DOI:** 10.3390/s16010029

**Published:** 2016-01-02

**Authors:** Yingyou Wen, Rui Gao, Hong Zhao

**Affiliations:** 1 College of Information Science and Engineering, Northeastern University, Shenyang 110819, China; yingyou_wen@163.com (Y.W.); hongzhao_neu@126.com (H.Z.); 2 Key Laboratory of Medical Image Computing of Ministry of Education, Northeastern University, Shenyang 110179, China

**Keywords:** wireless sensor networks, target tracking, generalized Kalman filter, neighborhood function, fuzzy

## Abstract

Moving target tracking in wireless sensor networks is of paramount importance. This paper considers the problem of state estimation for *L*-sensor linear dynamic systems. Firstly, the paper establishes the fuzzy model for measurement condition estimation. Then, Generalized Kalman Filter design is performed to incorporate the novel neighborhood function and the target motion information, improving with an increasing number of active sensors. The proposed measurement selection approach has some advantages in time cost. As such, if the desired accuracy has been achieved, the parameter initialization for optimization can be readily resolved, which maximizes the expected lifespan while preserving tracking accuracy. Through theoretical justifications and empirical studies, we demonstrate that the proposed scheme achieves substantially superior performances over conventional methods in terms of moving target tracking under the resource-constrained wireless sensor networks.

## 1. Introduction

A wireless network consisting of a large number of small sensors with low-power transceivers can be an effective tool for gathering data in a variety of environments [1]. These sensors are deployed at a cost much lower than traditional wired sensor systems, and have played significant roles in security and surveillance control, health care, and habitat monitoring in recent years [2,3,4]. Keeping the cost and size of these sensors small, they are equipped with low computation capability, small amounts of memory, and limited energy resources. Wireless sensor networks (WSN) must rely on these sensors and collaborative signal processing to dynamically manage node resources and effectively process distributed information [5,6,7]. Along this direction, moving target tracking will be considered in WSN.

Moving target tracking continuously reports the position of the target in terms of its coordinates to a fusion center or a central base station. It transfers one piece of sensor data to its coordinator, and determines its physical location relative to other neighboring coordinators [8]. Moving objects tracking methods are often considered with different criteria. These methods are categorized as tree-based target tracking, cluster-based target tracking and prediction-based target tracking [9]. In tree-based target tracking, nodes in wireless network are organized in a hierarchical tree structure or represented as a graph structure. Zhang *et al.* [10] proposed the concept of dynamic convoy tree-based collaboration, and formalized it as the moving objective optimization problem which needed to find a convoy tree sequence with high tree coverage. Gui *et al.* [11] proposed a collaborative messaging scheme that woke up and shut down the sensor nodes with spatial and temporal preciseness. This study quantized the trade-off between power conservation and quality of surveillance while presented guidelines for efficient deployment of sensor nodes in target tracking applications. Mehta *et al.* [12] formalized the location privacy issues in sensor networks under this strong adversary model and computed a lower bound on the communication overhead needed for achieving a given level of location privacy. Alaybeyoglu *et al.* [13] proposed one approach to awake nodes, which formed look-ahead clusters along the predicted trajectory to decrease the probability of missing the target. In cluster-based target tracking, the cluster member nodes identify the target and send the data to cluster heads. Cluster heads collect all data from members, determine target location and send the data to the sink node. Younis *et al.* [14] developed the approach to dynamically adapt the network topology within the cluster, minimized the energy consumption for communication, and extended the life of the network while achieving acceptable performance for data transmission. Bernabe *et al.* [15] proposed a novel cluster selection method based on similar ideas and tools using the camera activation mechanism, which was capable of accurately tracking multiple faces in real-time applications. Jiang *et al.* [16] presented the probability-based prediction and sleep scheduling protocol (PPSS) to improve energy efficiency. The approach designed one target prediction method based on kinematics and probability. Teng *et al.* [17] described the state evolution model which was employed to describe the dynamical system with neither prior knowledge of the target moving manner nor precise location information of the sensors. The joint posterior distribution of the parameters was updated online by incorporating the incomplete and inaccurate measurements between the target and each of the sensors into a Bayesian filtering framework. Most existing approaches in sensor networks concentrated on finding efficient ways for transmitting the data report to the data center, and not much work has been done on how to detect operative sensor nodes and generate robust and reliable data in an efficient way. Prediction-based methods, with prediction the target trajectory and its next location, only activate special nodes of network for tracking and rest of nodes remain in sleep mode for energy saving. Xu *et al.* [18] addressed the energy management issue in sensor networks and proposed a prediction-based energy saving scheme to reduce the energy consumption for object tracking under acceptable conditions. Bhuiyan *et al.* [19] proposed a set of fully distributed tracking algorithms, which answered queries on whether a target remains in localized geographic routing (LGR). Deldar *et al.* [20] used two parameters, distances from predicted location and remaining energy of nodes, for selection sensor nodes for tracking. Mazuelas *et al.* [21] detected the presence of non-line-of-sight (NLOS) propagation and estimated the ratio of the measurements coming from NLOS propagation. The approach identified the accurate measurements to achieve wireless location systems. Although the prediction-based approaches track the moving objects more accurately, predicted structures result in high-energy consumptions. There are two major shortcomings to these methods: (1) the sensor nodes takes much computation to measure the selections and (2) the number of nodes are determined by the capabilities of the fusion center. For those reasons, our focus here is on measurement condition estimation and tracking algorithms that are designed specifically based on neighborhood function.

Motivated by the above scenarios and concerns, the design of our approach relies on the prediction structure. In this paper, we propose a linear dynamic system with multiple sensors to track the target and monitor its surrounding area. The task is to extend the WSN lifespan without compromising the desired tracking accuracy. First, the paper establishes the fuzzy model for measurement condition estimation. Then, Generalized Kalman Filter (GKF) design is performed to incorporate the novel neighborhood function and the target motion information, improving with an increasing number of active sensors. The proposed measurement selection approach has some advantages in time cost. As such, if the desired accuracy has been achieved, the parameter initialization for optimization can be readily resolved, which maximizes the expected lifespan while preserving tracking accuracy.

The rest of the paper is organized as follows. In Section 2, we present the state estimation problem. Energy-efficient moving target tracking algorithms are proposed in Section 3. We conduct performance evaluations by simulation comparisons in Section 4. Finally, some conclusions and future work are given in Section 5.

## 2. Problem Formulation

We assume that *n* identical sensor nodes are densely deployed over a 2D area using a uniform random distribution. All nodes can only get connectivity information in neighbor nodes and measure Received Signal Strength (RSS) [22] in sensor nodes. All communication links among neighbor nodes are symmetric. These nodes have the same communication radius, denoted by *r*. These nodes are connected, that is to say at least one routing path exists between any pair of nodes. Note that two nodes are neighbor nodes if and only if $d_{ij}⩽r$, the target will be detected and the distance will be estimated. We use $M_{i}=\left\{j|j\neq i\;and\;d_{ij}⩽r\right\}$ to denote the set of neighbor nodes of node *i*. The network consists of *n* nodes, and there are *m* anchor nodes and n-m unknown nodes among them. Anchor nodes are aware of their coordinates. For convenience, the problem on transmission delay and packet loss is ignored. Specifically, we consider an *L*-sensor linear dynamic system [23].

(1) $$z_{n}\left(k\right)=h_{n}\left(x\left(k\right)\right)+v_{n}\left(k\right),n\in L_{k}$$

where $x\left(k\right)$ is the target position, and $z_{n}\left(k\right)$ is the measurement of the *n*th sensor at time instant *k*. $h_{n}\left(x\left(k\right)\right)=∥x\left(k\right)-\rho_{n}∥$ is the sensor target distance at the *k*th timestep, $v_{n}\left(k\right)$ is the observation noise at the *n*th sensor, and $\rho_{n}$ is the location of sensor node.

In the practical application, the system that provides such detecting measurements at the *k*th timestep is shown in the matrix form [24].

(2) $$\begin{array}{cc} Z_{k}= & H_{k}\left(X_{k}\right)+V_{k}\\ = & \left[\begin{array}{c} h_{1}\left(x\left(k\right)\right)\\ h_{2}\left(x\left(k\right)\right)\\ \vdots \\ h_{k}\left(x\left(k\right)\right) \end{array}\right]+\left[\begin{array}{c} v_{1}\left(k\right)\\ v_{2}\left(k\right)\\ \vdots \\ v_{k}\left(k\right) \end{array}\right] \end{array}$$

(3) $$L_{k}=\left\{n|∥x\left(k\right)-\rho_{n}∥⩽r,1⩽n⩽N\right\}$$

Here, the subscripts of *h* and *v* in Equation (2) refer to the indices within $L_{k}$ rather than node indices among all *N* sensor nodes, and then the covariance matrix of $V_{k}$ is

(4) $$V_{k}=diag{\left(\sigma^{2},\cdots ,\sigma^{2}\right)}_{l_{k}*l_{k}}$$

The transition Equation (2) as a discrete time dynamic state describes the motion of the moving target.

(5) $$X_{k}=FX_{k-1}+GW_{k-1}$$

where $X_{k}$ is a 4D vetor, consisting of the position vector $x\left(k\right)$. For the tracking application, one has

(6) $$F=\left[\begin{array}{cccc} 1 & \Delta & 0 & 0\\ 0 & 1 & 0 & 0\\ 0 & 0 & 1 & \Delta \\ 0 & 0 & 0 & 1 \end{array}\right]$$

(7) $$G=\left[\begin{array}{cc} \Delta^{2}/2 & 0\\ \Delta & 0\\ 0 & \Delta^{2}/2\\ 0 & \Delta \end{array}\right]$$

Here, Δ is the sampling time interval, and $W_{k-1}={\left[w_{x},w_{y}\right]}^{T}$ is a Gaussian random vector with zero mean. To facilitate collaborated sensor signal processing, sensor nodes within the sensing range of the target dynamically form the cluster. All other sensor nodes within the cluster will transmit their observations to the cluster head.

## 3. Proposed Approach

**Figure 1:**
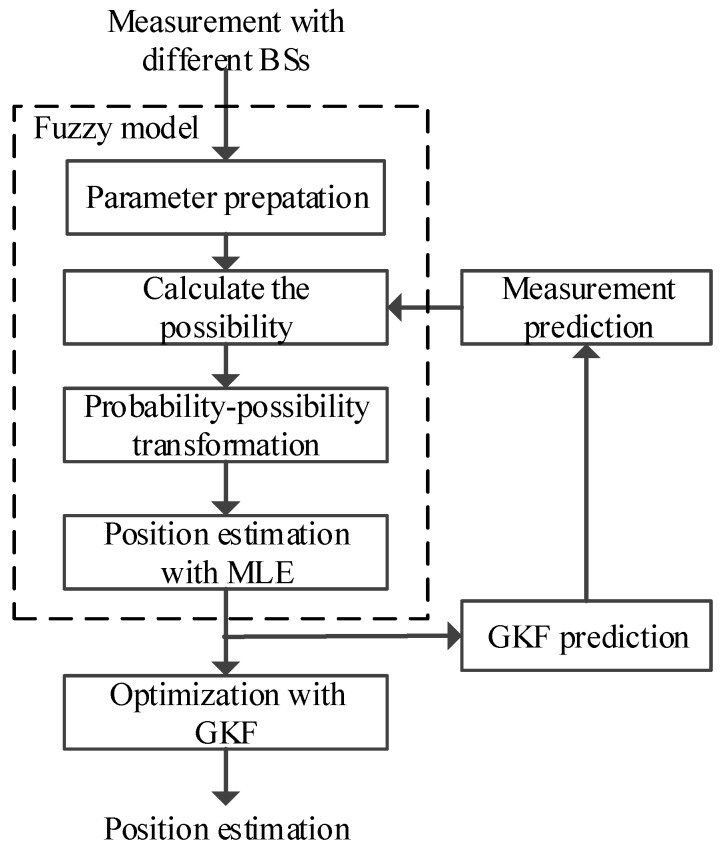
The proposed tracking approach.

The details of the proposed approach are described in Figure 1, which contains three main steps: (1) measurement selection based on fuzzy modeling; (2) position estimation with neighborhood function; and (3) optimization with GKF. Firstly, the measurement possibility is calculated based on the probability-possibility transformation. Then, the measurements with high possibility and low possibility are considered as the *L*-sensor linear dynamic system measurements for position calculation via neighborhood function. Finally, GKF is utilized to produce the optimization of smoothed position estimates.

### 3.1. Measurement Selection

To perform such a transformation, some prior information is required, and it is considered as an approximation of the optimal transformation. In [25], the paper describes the possibility of specific type of measurement from the physical sensor using the probability-possibility transformation. Applying this theory, fuzzy modeling of measurements associated with each base station (BS) is established to calculate the possibility that the corresponding measurement is taken under linear dynamic system. We devote the function $\pi \left(x\right)$ as the probability-possibility transformation. When $\left|x-x_{c}\right|$ is less than $\left(x_{\varepsilon }-x_{c}\right)$, the function value is $1-\left(1-\varepsilon \right)\left|x-x_{c}\right|/\left(x_{\varepsilon }-x_{c}\right)$; when $\left|x-x_{c}\right|$ is greater than $\left(x_{\varepsilon }-x_{c}\right)$, $\pi \left(x\right)$ is equal to zero; otherwise, the value is *ε*. The parameters such as $x_{c}$, *x*, $x_{\varepsilon }$ are described in Table 1 for different measurement noise laws.

The mean $x_{m}$ can be calculated by

(8) $$x_{m}=\sqrt{{\left(x_{k/k-1}-x_{BS}\right)}^{2}+{\left(y_{k/k-1}-y_{BS}\right)}^{2}}$$

where $x_{m}$ Raman and *σ* represent the mean and the standard deviation of the measurement data, respectively.

**Table 1:** The parameters of the probability-possibility transformation.

The Law	$x^{c}$	*x*	$x_{\epsilon }$	*ϵ*
Gaussian Law	$x_{m}$	$x_{m}+\sigma$	$x_{m}+\sigma$	0.12
Exponent Law	$x_{m}$	$x_{m}+\sigma$	$x_{m}+\sigma$	0.13
Triangular Law	$x_{m}$	$x_{m}+\sigma$	$x_{m}+\sigma$	0.11
Uniform Law	$x_{m}$	$x_{m}+\sigma$	$x_{m}+\sigma$	0

### 3.2. Position Estimation with Neighborhood Function

**Figure 2:**
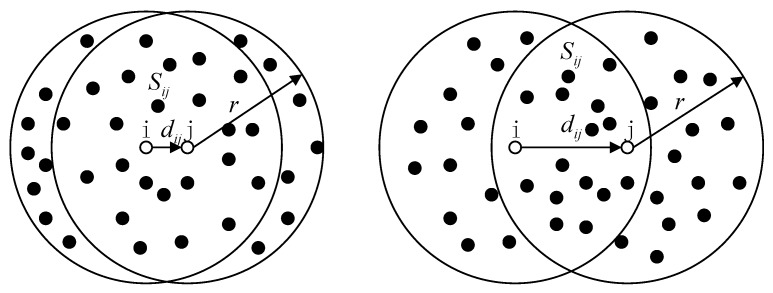
The distance model between neighbor nodes.

We propose a novel distance estimation method only using connectivity information and geometric features between neighbor nodes. Figure 2 shows the distance model between two neighbor nodes *i* and *j*. The black solid points are other neighbor nodes of nodes *i* and *j*. We can observe that the distance $d_{ij}$ between node *i* and its neighbor node *j* determine the size of the intersection area denoted by $S_{ij}$, and $S_{ij}$ is inversely proportional to $d_{ij}$. $S_{ij}$ can be calculated as follows.

(9) $$S_{ij}=2r^{2}arccos\left(\frac{d_{ij}}{2r}\right)-d_{ij}\sqrt{r^{2}-\frac{d_{ij}^{2}}{4}}$$

The ratio of $S_{ij}$ and the communication area *S* of node *i* can be calculated by

(10) $$\frac{S_{ij}}{S}=\frac{S_{ij}}{\pi r^{2}}=\frac{2}{\pi }arccos\left(\frac{d_{ij}}{2r}\right)-\frac{d_{ij}}{\pi r}\sqrt{1-{\left(\frac{d_{ij}}{2r}\right)}^{2}}$$

Equation (10) can also be written as

(11) $$\begin{array}{cc} & y=\frac{S_{ij}}{\pi r^{2}},x=\frac{d_{ij}}{2r}\\ & y=\frac{2}{\pi }arccos\left(x\right)-\frac{2}{\pi }x\sqrt{1-x^{2}} \end{array}$$

Using Taylor series expansion, $arccos\left(x\right)$ and $x\sqrt{1-x^{2}}$ can be written as

(12) $$\begin{array}{cc} arccos\left(x\right) & =\frac{\pi }{2}-x-\frac{1}{6}x^{3}-\frac{3}{40}x^{5}-\cdots \\ x\sqrt{1-x^{2}} & =x-\frac{x^{3}}{2}-\frac{1}{8}x^{5}-\cdots \end{array}$$

According to Equations (11) and (12), Equation (10) can be written as

(13) $$\frac{S_{ij}}{\pi r^{2}}=1-\frac{2}{\pi }\left(\frac{d_{ij}}{r}\right)+\frac{1}{12\pi }{\left(\frac{d_{ij}}{r}\right)}^{3}+\frac{1}{320\pi }{\left(\frac{d_{ij}}{r}\right)}^{5}$$

The value of $d_{ij}$ is the distance between neighbor nodes, and the value range of $d_{ij}/r$ should be $0⩽d_{ij}/r⩽1$. From Equation (13), we can observe $S_{ij}/r^{2}$ is mainly determined by $2*\left(d_{ij}/r\right)/\pi$, so it is an approximate linear function relationship between $S_{ij}/\pi r^{2}$ and $d_{ij}/r$. When $d_{ij}/r$ is 0, $S_{ij}/r^{2}$ is 1. When $d_{ij}/r$ is 1, $S_{ij}/r^{2}$ is 0.391. We can get the linear function as

(14) $$\frac{d_{ij}}{r}=\frac{1}{0.609}\left(1-\frac{S_{ij}}{\pi r^{2}}\right)$$

As we know, the density of sensor nodes is high for range-free wireless sensor networks. Figure 2 describes that the area is direct ratio to the number of nodes, so $S_{ij}$ can be estimated by

(15) $$S_{ij}\approx \sigma \cdot \frac{N_{ij}}{N_{i}}\cdot \pi r^{2}$$

where $N_{ij}=\left|M_{i}\cap M_{j}\right|+2$ is the number of nodes within the intersection area $S_{ij}$ , and $N_{i}=M_{i}+1$ is the number of nodes within the communication range of node *i*. *σ* is a correction parameter to make Equation (15) more accurate and the concrete value will be given in the following test. In practice, $N_{ij}$ and $N_{i}$ can be easily obtained by exchanging the neighbor information between nodes *i* and *j*. Finally, we get an important equation from Equations (14) and (15)

(16) $$D_{{NDR}_{ij}}=\frac{d_{ij}}{r}=\frac{1}{0.609}\left(1-\sigma \cdot \frac{N_{ij}}{N_{i}}\right)$$

where $D_{{NDR}_{ij}}$ is used to denote the neighborhood distance relationship (NDR) from node *i* to its neighbor node *j*. However, because all nodes are randomly deployed, it is not uncommon that $N_{i}\;\neq \;N_{j}$. That is to say it is not uncommon that $D_{{NDR}_{ij}}\neq D_{{NDR}_{ji}}$. Since the bigger $N_{i}$ is, the more accurate the estimated $S_{ij}$ in Equation (16) is, we use this as follows to estimate $D_{NDR}$ between neighbor nodes *i* and *j* in this paper

(17) $$D_{{NDR}_{ij}}=D_{{NDR}_{ji}}=\frac{d_{ij}}{r}=\frac{1}{0.609}\left(1-\sigma \cdot \frac{N_{ij}}{max\left(N_{i},N_{j}\right)}\right)$$

where the function $max\left(N_{i},N_{j}\right)$ is used to take the maximum of $N_{i}$ and $N_{j}$. From Equation (17) we estimate the distance between neighbor nodes $d_{ij}$ as follows

(18) $$\tilde{d_{ij}}=r\cdot D_{{NDR}_{ij}}$$

However, $D_{{NDR}_{ij}}$ is an estimated value and not accurate enough, and the estimated distance $d_{ij}$ is also not accurate. Getting the $D_{{NDR}_{ij}}$ between neighbor nodes, we use the Floyd–Warshall path algorithm to calculate the shortest NDR-path which is minimal the one with the minimum value of all NDR-path between two anchor nodes. Then, the paper computes an NDR correction factor $\lambda_{NDR}$ as follows

(19) $$\lambda_{NDR}=\frac{\sum_{k=1}^{m}\sum_{s=1}^{m}d_{ks}}{\sum_{k=1}^{m}\sum_{s=1}^{m}min\;D_{{NDR}_{ks}}}$$

where $d_{ks}$ is the Euclidean distance between anchor nodes *k* and *s*. *m* is the number of anchor nodes. Finally, we estimate the distance between two neighbor nodes as follows

(20) $${\tilde{d}}_{ij}={\tilde{d}}_{ji}=\lambda_{NDR}*D_{{NDR}_{ij}}$$

Ignoring the constant in Equation (20), the log likelihood function of all the distance measurements can be written as

(21) $$f\left(x_{k},y_{k}\right)=\sum_{i=1}^{N}\frac{{\left(z_{i,j}-\left(d_{i,j}+\mu \right)\right)}^{2}}{\sigma^{2}}$$

Applying the Maximum likelihood estimator produces the mobile position estimate as follows

(22) $${\left(x_{k},y_{k}\right)}_{MLE}=argminf\left(x_{k},y_{k}\right)$$

### 3.3. Optimization with Generalized Kalman Filter

The traditional Kalman filter can be viewed as a recursive stochastic algorithm. For the large application, GKF is more efficient than traditional Kalman filter. When getting the intermediate position estimation, we compute better position estimates by the GKF. The intermediate estimate $z_{k}$ can be described as a linear equation of the target state:

(23) $$\begin{array}{cc} z\left(k\right)= & Hx\left(k\right)+v\left(k\right) \end{array}$$

(24) $$\begin{array}{cc} x\left(k\right)= & Ax\left(k-1\right)+w\left(k-1\right) \end{array}$$

(25) $$\begin{array}{cc} H= & \left[\begin{array}{ccc} 1 & 0 & 0\\ 0 & 1 & 0 \end{array}\right] \end{array}$$

where $w\left(k-1\right)$ is a zero-mean white Gaussian noise vector with covariance matrix. In [26], the noise $v\left(k\right)$ is white Gaussian with zero mean. Th implementing the GKF is performed according to the following

(26) $$\begin{array}{cc} \hat{x}\left(k/k-1\right)= & A\hat{x}\left(k-1/k-1\right) \end{array}$$

(27) $$\begin{array}{cc} P\left(k/k-1\right)= & AP\left(k-1/k-1\right)A^{T}+Q\left(k-1\right) \end{array}$$

(28) $$\begin{array}{cc} P\left(k/k\right)= & \left[I-K\left(k\right)H\right]P\left(k/k-1\right) \end{array}$$

(29) $$\begin{array}{cc} \hat{x}\left(k/k\right)= & \hat{x}\left(k/k-1\right)+K\left(k\right)*\left[z\left(k\right)-H\hat{x}\left(k/k-1\right)\right] \end{array}$$

(30) $$\begin{array}{cc} K\left(k\right)= & P\left(k/k-1\right)H^{T}{\left[R\left(k\right)+HP\left(k/k-1\right)H^{T}\right]}^{-1} \end{array}$$

where $K\left(k\right)$ is the Kalman gain, $\hat{x}\left(k/k\right)$ is the state update including the desired position estimate at time instant *k*, and $P\left(k/k-1\right)$ and $P\left(k/k\right)$ are the state covariance predictions.

## 4. Simulation Results

In this section, we evaluate the performance of tracking through extensive simulations and provide more insight into the tracking issues from simulation perspectives. We consider a 500 m × 500 m deployment field with 300 sensor nodes. All sensor nodes are randomly distributed, and these nodes can get connectivity information to measure Received Signal Strength (RSS) or other information.

### 4.1. Comparison of Estimated Distance Error

Estimated distance error (EDE) is the average absolute difference between the estimated distance and corresponding real inter-node distance. In this section, we compare the values of EDE on three algorithms, including the method based on distance vector in hops (DV-HOP) [27], LGR and the proposed approach.

(31) $$\begin{array}{cc} EDE= & \frac{1}{r\sum_{i=m+1}^{n}\left|M_{i}\right|}\sum_{i=m+1}^{n}\sum_{j\in M_{i}}\left|d_{ij}-{\tilde{d}}_{ij}\right|\times 100\%\\ d_{ij}= & \sqrt{{\left(x_{i}-x_{j}\right)}^{2}+{\left(y_{i}-y_{j}\right)}^{2}} \end{array}$$

where $M_{i}$ is the number of neighbor nodes of unknown node *i*, $d_{ij}$ is the real distance between neighbor nodes, and ${\hat{d}}_{ij}$ is the estimated distance generated by the distance estimation method. $\left(x_{i},y_{i}\right)$ and $\left(x_{j},y_{j}\right)$ respectively denote the true positions of nodes *i* and *j*. In order to make the test more comprehensive, we test the impact of different communication range and number of anchor nodes on the EDE. All results are averaged over 100 different network deployments.

**Figure 3:**
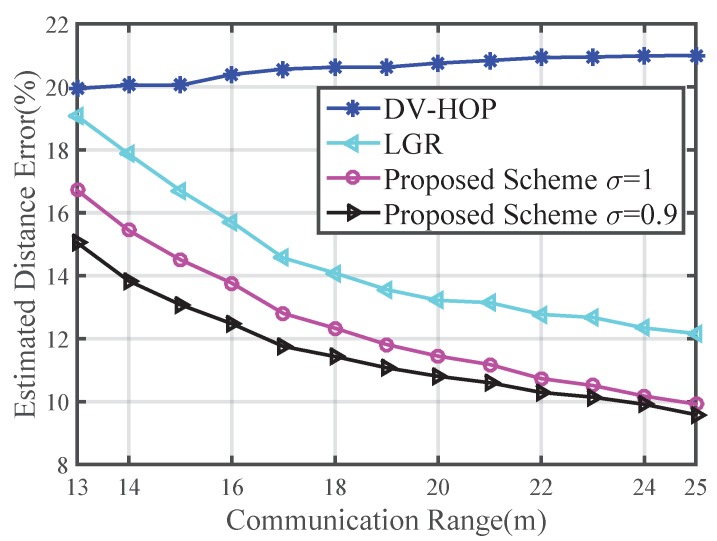
Impact of the communication range on EDE.

The impact of communication range on EDE is shown in Figure 3. We set the number of anchor nodes as 20. The variation of communication range is from 13 m to 25 m. If the communication range is smaller than 13 m, sometimes the network is not connected. The EDE of DV-HOP changes little as the communication range increases because increased communication range confuses the nodes when hop count is carried out. LGR and our approach decrease as the communication range increases. The result shows that the distance estimation method in this paper is always better than those of the DV-HOP and LGR algorithms.

**Figure 4:**
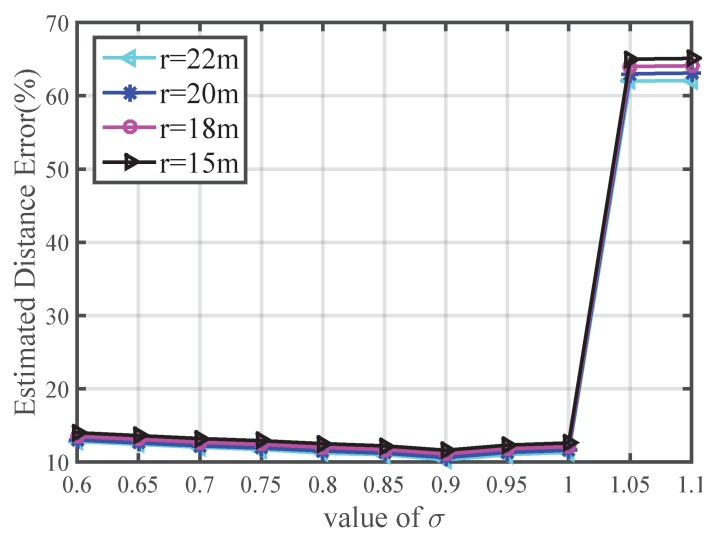
Impact of the correction parameter *σ* on EDE.

We also test the impact of the correction parameter *σ* in Equation (17) on the distance estimation method in this paper. Figure 4 shows that the EDE value of the proposed approach increases when σ>1, and the EDE in this paper with σ=0.9 is always smaller. This verifies the effectiveness of the correction parameter *σ* and we set σ=0.9 in subsequent tests.

### 4.2. Comparison of the Averages of Root Mean Square Error

We set $\left[{\hat{x}}_{j}\left(k/k\right),{\hat{y}}_{j}\left(k/k\right)\right]$ as the corresponding position estimation at the *k*th time instant. The average of root mean square error (RMSE) is defined by [21].

(32) $$RMSE=\sqrt{\frac{1}{M}\sum_{j=1}^{M}\left[{\left({\hat{x}}_{j}\left(k/k\right)-x\left(k\right)\right)}^{2}+{\left({\hat{y}}_{j}\left(k/k\right)-y\left(k\right)\right)}^{2}\right]}$$

**Figure 5:**
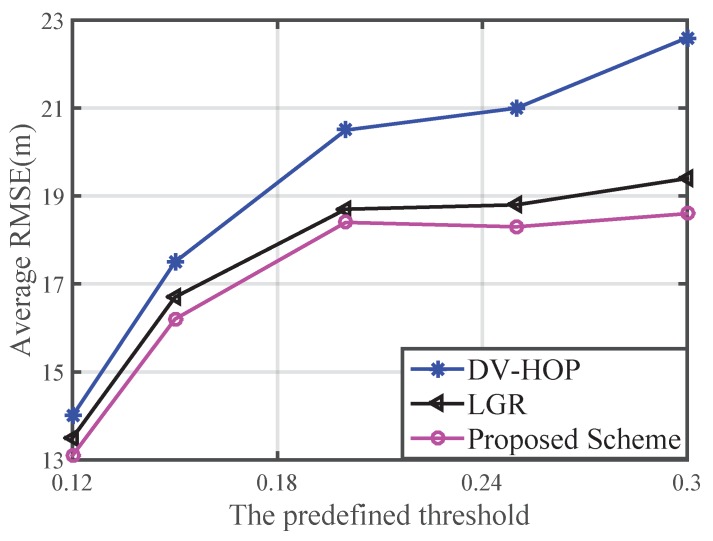
Average RMSE versus the predefined possibility threshold.

Here, *M* is the total number of Monte Carlo test. Figure 5 shows the performance in terms of average RMSE with respect to different predefined possibility thresholds. It can be seen that the average RMSE is smallest in simulation scenarios, when the threshold is equal to 0.12. The average RMSE on this paper is lower than those of the DV-HOP and LGR algorithms.

## 5. Conclusions and Future Work

In this article, we have described the implementation of energy efficient moving target tracking in wireless sensor networks where measurements from a subset of sensors are employed at each time step. The three-step moving target tracking scheme is proposed to maximize the expected lifespan while preserving tracking accuracy. A fuzzy modeling method is developed under *L*-sensor linear dynamic system for measurement selection. After analyzing the relationship between distance and intersection area of neighborhood nodes, we describe the novel neighborhood function as position estimation. The position optimization is smoothed by using the linear GKF to produce better positioning performance. The position prediction from the GKF is utilized for parameter initialization in the probability–possibility transformation. Numerical experiments show that the proposed approach outperforms the existing algorithms in terms of EDE and average RMSE. In the future, we will implement a real world wireless sensor network to track moving targets.
